# The Effect of Technology-Based Interventions on Pain, Depression, and Quality of Life in Patients With Cancer: A Systematic Review of Randomized Controlled Trials

**DOI:** 10.2196/jmir.4009

**Published:** 2015-03-13

**Authors:** Stephen O Agboola, Woong Ju, Aymen Elfiky, Joseph C Kvedar, Kamal Jethwani

**Affiliations:** ^1^Partners Healthcare Center for Connected HealthBoston, MAUnited States; ^2^Massachusetts General HospitalBoston, MAUnited States; ^3^Harvard Medical SchoolBoston, MAUnited States; ^4^Medical Research Institute, College of MedicineDepartment of Obstetrics and GynecologyEwha Womans UniversitySeoulRepublic Of Korea; ^5^Dana-Farber Cancer InstituteBoston, MAUnited States

**Keywords:** telehealth, connected health, cancer, telephone, pain, depression, quality of life, systematic review, randomized controlled trials

## Abstract

**Background:**

The burden of cancer is increasing; projections over the next 2 decades suggest that the annual cases of cancer will rise from 14 million in 2012 to 22 million. However, cancer patients in the 21st century are living longer due to the availability of novel therapeutic regimens, which has prompted a growing focus on maintaining patients’ health-related quality of life. Telehealth is increasingly being used to connect with patients outside of traditional clinical settings, and early work has shown its importance in improving quality of life and other clinical outcomes in cancer care.

**Objective:**

The aim of this study was to systematically assess the literature for the effect of supportive telehealth interventions on pain, depression, and quality of life in cancer patients via a systematic review of clinical trials.

**Methods:**

We searched PubMed, EMBASE, Google Scholar, CINAHL, and PsycINFO in July 2013 and updated the literature search again in January 2015 for prospective randomized trials evaluating the effect of telehealth interventions in cancer care with pain, depression, and quality of life as main outcomes. Two of the authors independently reviewed and extracted data from eligible randomized controlled trials, based on pre-determined selection criteria. Methodological quality of studies was assessed by the Cochrane Collaboration risk of bias tool.

**Results:**

Of the 4929 articles retrieved from databases and relevant bibliographies, a total of 20 RCTs were included in the final review. The studies were largely heterogeneous in the type and duration of the intervention as well as in outcome assessments. A majority of the studies were telephone-based interventions that remotely connected patients with their health care provider or health coach. The intervention times ranged from 1 week to 12 months. In general, most of the studies had low risk of bias across the domains of the Cochrane Collaboration risk of bias tool, but most of the studies had insufficient information about the allocation concealment domain. Two of the three studies focused on pain control reported significant effects of the intervention; four of the nine studies focus on depression reported significant effects, while only the studies that were focused on quality of life reported significant effects.

**Conclusions:**

This systematic review demonstrates the potential of telehealth interventions in improving outcomes in cancer care. However, more high-quality large-sized trials are needed to demonstrate cogent evidence of its effectiveness.

## Introduction

The burden of cancer is increasing globally; projections over the next two decades suggest that the annual cases of cancer will rise from 14 million in 2012 to 22 million [1]. Cancer is the leading cause of death worldwide and the second leading cause of death in the United States [1,2]. Encouragingly, cancer patients in the 21st century are living longer due to a combination of early detection, availability of novel therapeutic regimens, and improved supportive care. According to the National Cancer Institute, the 5-year survival rate for all cancers increased significantly from about 48.7% in 1975 to about 68.5% in 2006 in the United States [2]. Despite these notable improvements in cancer outcomes, many patients experience physical and/or emotional distress, resulting from complex interplays between the disease process and treatment modalities, which significantly impact quality of life [3,4]. In this context, extended longevity has necessarily prompted a growing focus on better defining, capturing, and maintaining health-related quality of life (HR-QOL).

An increasingly popular model for delivering supportive care for patients with cancer is telehealth or other terminologies including connected health, eHealth, mHealth, that are used to describe health care delivery that leverages technology. Telehealth offers patients the opportunity for long-term home monitoring, health education and coaching, behavioral modification, sharing health information with care providers, and timely feedback. It has been largely employed in the management of chronic disease such as diabetes, hypertension, and heart failure [5-10]. Nowadays, with many patients with cancer living longer, it is increasingly being used to engage patients with cancer. Over the last decade, a growing body of studies regarding the application of telehealth in cancer care has been published. Some of the common applications in cancer care include management of pain, cancer-related psychological effects, and overall use to improve quality of life. However, the evidence of its effectiveness in cancer care is still not solid due to difficulty in designing or implementing non-biased randomized controlled trials (RCT) exploring its true effect. For this reason, there is a dearth of published systematic reviews or meta-analyses that summarize this topic. In this study, we evaluate the effect of telehealth on pain, depression, and quality of life in cancer patients via a systematic review of RCTs.

## Methods

### Literature Search

We first searched PubMed, EMBASE, Google Scholar, CINAHL, and PsycINFO in July 2013 for prospective RCTs evaluating telehealth in cancer care regarding pain, depression, and quality of life. The search was updated in January 2015.The keywords were as follows: “neoplasms [MeSH]”, “cancer” and “Remote Consultation [Mesh]”, “mHealth”, “connected health”, “text messaging”, “telemedicine”, “telehealth”, “ehealth”, “telephone therapy”, “teleconsultation”, “mobile technology”, “telecare”, “Internet”, “digital health”, “mobile phone*”, “smartphone”, “apps”, and “mobile application”.

### Selection Criteria

We included RCTs that met all of the following criteria: reported the effect of telehealth on pain, depression, or quality of life in cancer patients. If data were duplicated or shared in more than one study, the last published or more comprehensive study was included in the analysis.

### Selection of Relevant Studies

Based on the pre-determined selection criteria, 2 authors (JW, SA) independently selected all trials retrieved from the databases and bibliographies. Disagreements between evaluators were resolved by discussion.

### Assessment of Methodological Quality

The methodological quality of included studies was assessed by the Cochrane Collaboration’s risk-of-bias tool [11], a commonly used tool to report the risk of bias in individual studies included in systematic reviews. The tool assesses several internal validity domains, which include sequence generation, allocation concealment, blinding of study participants, personnel and outcome assessors, incomplete outcome data, selective reporting, and other sources of bias. We classified each of these domains as being at high, low, or unclear risk of bias. Data were entered into Review Manager 5.3, and a risk of bias graph was generated for all included studies.

## Results

### Overview

**Figure 1:**
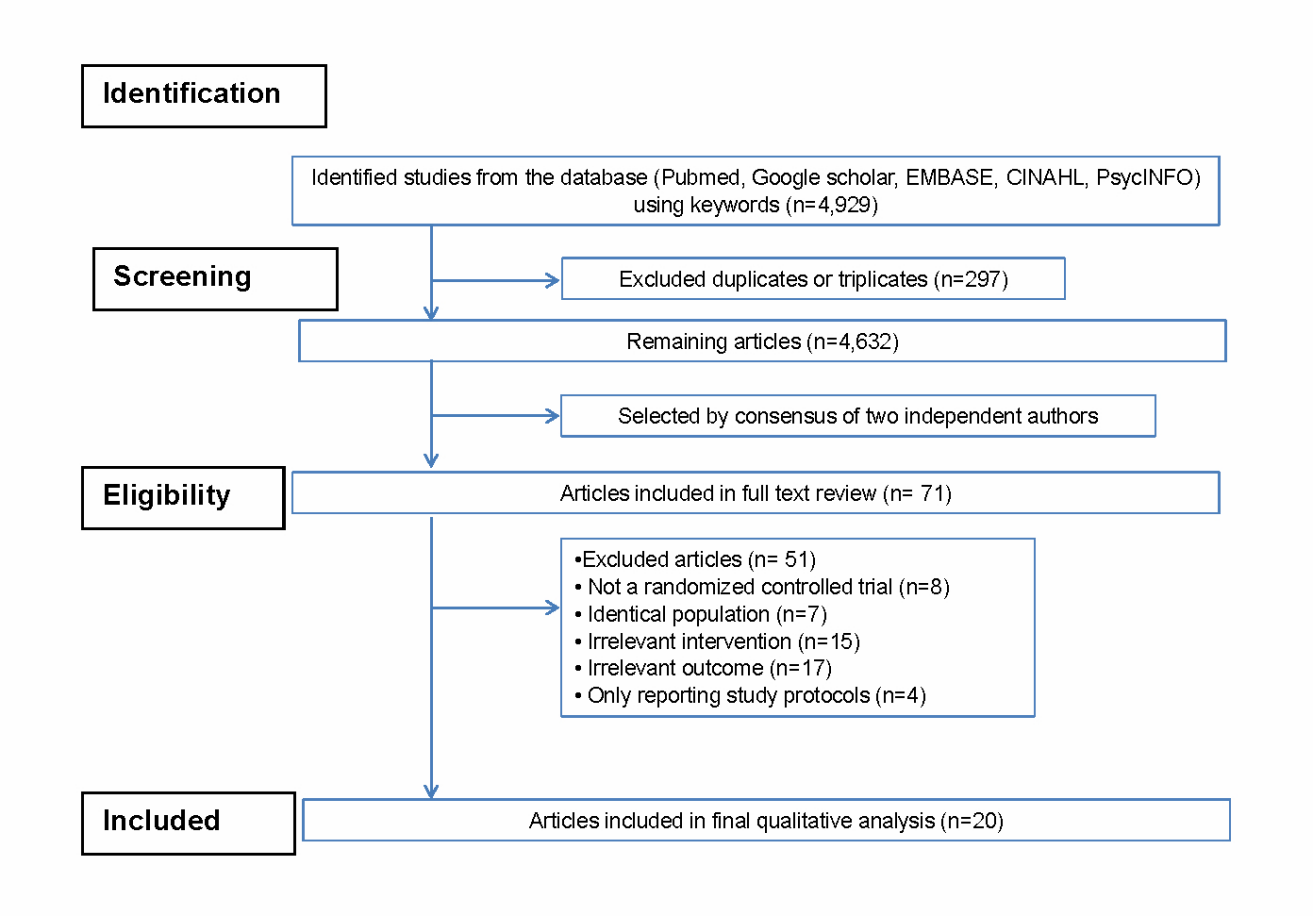
Flow diagram depicting the systematic review process.

Figure 1 depicts a flow diagram of how we identified relevant clinical trials included in this review. Using the above-mentioned keywords, a total of 4929 articles were identified from the literature search of five databases, that is, PubMed, EMBASE, Google Scholar, CINAHL, and PsycINFO. After excluding 297 duplicated articles, 2 authors independently reviewed and excluded an additional 4561 articles that did not satisfy the pre-determined selection criteria based on each article’s title and abstract. We reviewed the full texts of the remaining 71 articles and excluded 51 articles because of the following reasons: identical trials with the same population (n=7), nonrandomized studies (n=8), trials not related to the subject (intervention/outcome) of this study (n=32), and trials reporting only the study protocol (n=4). A total of 20 trials were included in the final analysis [12-31]. Since the studies included in this review are RCTs with comparator groups, we report only between-group effect estimates. Pre- and post measures within groups were not considered.

### Characteristics of Included Studies

Table 1 shows the general characteristics of the 20 trials. Eight (40%) of the 20 studies focused on improving quality of life [16,18,19,22,24,26,28,31], another nine (45%, 9/20) on improving depression outcome (Sherman et al actually focused on psychological well-being) [12,15,17,20,21,25,27,29,30], two (10%, 2/20) on improving pain control [13,14], and one (5%, 1/20) has both pain intensity and depression as main outcomes [23]. The Functional Assessment of Cancer Therapy (FACT) was the most (62.5 of all studies with HR-QOL as main outcome) commonly used measure of quality of life. The Center for Epidemiologic Studies Depression Scale (CES-D) and the Hospital Anxiety and Depression Scale (HADS) were the most commonly used measure for depression and the Brief Pain Inventory most commonly (66.7% of all studies with pain intensity as main outcome) used to evaluate pain outcomes. The studies were published over a period of 9 years from 2006-2014.

**Table 1:** Characteristics of randomized trials included in the systematic review on telehealth for cancer patients^a^.

Author, year, country	Technology	Participants	Objectives	Intervention	Comparator	Intervention time
Badger, [12] 2012, USA	Telephone	70 breast cancer patients and their supportive partners (SPs)	To evaluate the efficacy of two telephone-delivered interventions in improving quality of life among Latinas with breast cancer and their family members or friends	Telephone interpersonal counseling delivered by trained interventionist	Telephone health education delivered by trained professionals	8 weeks: eight weekly sessions for patients and four sessions every other week for SPs
Borosund [27] 2014, Sweden	Internet	167 breast cancer patients	To evaluate the effect of the components of a Web-based support tool on symptom distress, anxiety and depression	Two intervention arms: (1) Internet-based patient-provider communication (IPPC) tool, (2) Webchoice + IPPC. Webchoice facilitates symptom monitoring, self-management and communication with other patients	Usual standard of care at the hospital of treatment	6 months
Duffecy, [] 2012, USA	Internet	31 patients with any cancer	To evaluate the feasibility of a Web-based intervention in increasing adherence to the intervention and efficacy in reducing symptoms of depression in post cancer treatment survivors	Individual Internet Intervention +Internet Support Group (ISG). ISG included a discussion board and features to enhance supportive accountability	Individual Internet Intervention is a self-management program, based on cognitive behavioral principles, for the treatment of depression	8 weeks
Freeman, [28] 2014, USA	Video-conference	118 breast cancer survivors	To evaluate the effect of an imagery-based group intervention on quality of life in breast cancer survivors	Two intervention groups with five 4-hr weekly group session delivered by trained professionals via live sessions or video-conferencing plus weekly telephone calls	Wait-list controls	3 months
Gotay, [25] 2007, USA	Telephone	305 breast cancer patients	To evaluate the effectiveness of a peer-delivered telephone support intervention on psychosocial outcomes in patients with a first recurrence of breast cancer	Telephone counseling/ information sessions delivered by trained peer counselors at a breast cancer advocacy organization	Standard care	4-8 sessions weekly with 1-2 calls per week for 1 month
Harrison, [19] 2011, Australia	Telephone	75 colorectal cancer (CRC) patients	To evaluate the effectiveness of a nurse-delivered telephone supportive intervention in reducing unmet supportive care needs, reducing health service utilization, and improving HR-QOL post- discharge from the hospital after surgery for CRC	CONNECT: post-surgery follow-up telephone calls delivered by an experienced colorectal cancer nurse who has undergone training in telephone communication	Usual care: follow- up appointment with a general practitioner and surgeon	5 calls over 6 months
Hawkins, [22] 2010, USA	Telephone and web	434 breast patients	To evaluate the mediating processes of two communication interventions to improve HR-QOL in patients with breast cancer	3 intervention groups: (1) Access to the Web-based comprehensive Health Enhancement Support System (CHESS), (2) Telephone-based Cancer information mentor, (3) CHESS + Cancer Information Mentor	Internet training and access	10 times over 6 months
Kim, [14] 2013, Korea	Telephone	108 patients with any solid-organ tumor	To evaluate the effectiveness of standardized education and telemonitoring in improving pain, distress, anxiety, depression, HR-QOL, and performance in outpatients with advanced cancers	Telemonitoring performed by an NP trained in pain management	Standardized pain education based on the WHO and NCCN pain control guidelines delivered by NP on the first visit	30 mins every day for 1 week
Kroenke, [23] 2010, USA	Telephone and Internet	405 cancer patients	To evaluate the effect of a telephone-based care management combined with automated symptom monitoring on depression and pain in patients with cancer	Telephonic care management by a nurse care manager combined with automated symptom monitoring (via interactive voice-recorded telephone calls or Web-based surveys)	Usual care provided by oncologists.	Follow-up calls and automated symptom monitoring staggered over 12 months
Lepore, [29] 2014, USA	Internet	184 breast cancer patients	To test the mental health benefits of two Internet support group (ISG) interventions in women with breast cancer	Pro-social Internet support group (ISG) which includes all features of the Standard-ISG plus tips on recognizing and responding to others’ need for support and participation in a breast cancer awareness outreach activity	Standard-ISG with weekly live 90-minutes chats facilitated by PhD level interventionist plus discussion board for asynchronous text communication	6 weeks
Livingston, [20] 2009, Australia	Telephone	571 male colorectal (CRC) and prostate cancer patients	To evaluate the psychological impact of a referral and telephone intervention, involving information and support, among men with CRC and prostate cancer	Cancer Helpline: telephone calls from cancer nurses to help patients address issues they may experience during cancer care. 2 intervention groups: (1) Active Referral—4: four outcalls, (2) Active Referral—1: one outcall.	Passive Referral: usual care which involved a specialist referral to the Helpline but contact was at the participant’s initiative	Active Referral—4: four outcalls staggered over 6 months post-diagnosis. Active Referral—1: outcall within 1 week of diagnosis.
Loprinzi, [18] 2011, USA	Telephone	25 breast cancer survivors	To evaluate the effect of a Stress Management and Resiliency Training (SMART) program for increasing resiliency and for decreasing stress and anxiety among breast cancer mentors who themselves were previously diagnosed with breast cancer	The SMART program: consisted of 3 parts: 2 small-group, 90-minute sessions teaching the SMART program; a brief individual follow-up session with a study investigator; and 3 follow-up telephone calls	Wait list group. Intervention delayed by 12 weeks.	12 weeks: telephone calls at 4-week intervals. Each call lasted approximately 15 minutes
Marcus, [21] 2009, USA	Telephone	304 breast cancer patients	To evaluate the effect of a telephone counseling program on psychosocial outcomes among breast cancer patients post-treatment	Usual care + Telephone Counseling program delivered by four Masters-level psychosocial oncology counselors	Usual care: booklet listing psychosocial and other social service and rehabilitation resources in their community for breast cancer	16 sessions delivered over a 12-month period. Each session lasted 45 mins
Nelson, [24] 2008, USA	Telephone	50 cervical cancer patients	To evaluate the feasibility of a psychosocial telephone counseling intervention designed for patients with cervical cancer on improving HR-QOL	Psychosocial telephone counseling intervention, delivered by a psychologist, designed to help women cope with the stressful events and feelings of distress associated with cervical cancer	Usual care	5 weeks: weekly session about 45 to 50 min in length + 1 month booster later
Park, [16] 2012, Korea	Telephone	48 breast cancer patients	To evaluate the effect of a psycho-educational support program on HR-QOL and symptom experience for women in the first year post-breast cancer treatment survivorship	Psychoeducation plus Standard care. The psychoeducational program consisted of individual face-to-face education using a participant handbook, telephone-delivered health-coaching sessions, and small-group meetings	Standard care from their medical team plus a short booklet on cancer care	12 weeks: 10-30 mins telephone coaching sessions every other week
Rustoen, [13] 2013, Norway	Telephone	179 cancer patients with bone metastasis	To evaluate the efficacy of PRO-SELF in decreasing pain intensity scores and increasing opioid intake in cancer patients.	PRO-SELF: Individualized pain management education delivered by oncology intervention nurses who visited patients in their homes at weeks 1, 3, and 6 and conducted telephone interviews at weeks 2, 4, and 5	Cancer pain management booklet plus home visits and nurse telephone interviews with the same frequency as patients in the intervention to monitor level of adherence with completing the pain diary	6 weeks
Ryhanen, [31] 2013, Finland	Internet	90 breast cancer patients	To evaluate the effect of the Breast Cancer Patient Pathway (BCPP) program on patients’ empowerment process. Specifically looking at quality of life, anxiety, and side-effects	Hospital standard of care plus the BCPP program - an Internet-based patient education tool to increase patients’ knowledge about breast cancer	Oral and written education materials according to hospital standards	Throughout the treatment period, average of 9 months
Sandgren, [26] 2006, USA	Telephone	218 breast cancer patients	To evaluate the effectiveness of two telephone-based interventions in improving mood and HR-QOL in patients with breast cancer	Telephone counseling including health education and emotional expression therapy delivered by oncology nurses	Standard care	5 weekly 30-minutes phone calls, with a 6th, follow-up call, made approx. 3 months later
Sherman, [17] 2012, USA	Telephone	249 breast cancer patients	To evaluate the effect of three technology-based interventions on physical, emotional, and social adjustment of women with early-stage breast cancer	3 intervention groups: (1) usual care + four phase-specific psychoeducational videos, (2) Usual care + four phase-specific telephone counseling sessions delivered by nurse interventionist, (3) usual care + phase-specific psycho-educational videos+ phase-specific telephone counseling sessions	Usual care was standardized across all sites according to national treatment protocols for the diagnosis and treatment of breast cancer.	Phase-specific: four phases of the breast cancer experience: diagnosis, post-surgery, adjuvant therapy and ongoing recovery
Stanton, [30] 2013, USA	Internet	88 breast cancer patients	To evaluate the effect of an Internet-based invention designed for chronicling the cancer experience and promoting communication	Project Connect Online: patients taught how to develop personalized website where they can journal their cancer experience and share content with their social networks	Waiting-list control	6 months

^a^HR-QOL: Health-related Quality of Life; CHESS: Comprehensive Health Enhancement Support System; WHO: World Health Organization; NP: nurse practitioner; SP: supportive partner; NCCN: National Comprehensive Cancer Network; CRC: colorectal carcinoma; SMART: Stress Management and Resiliency Training.

The majority (13/20, 65%) of the studies were conducted in the United States. The other countries represented include Australia (2/20, 10%), South Korea (2/20, 10%), Sweden (1/20, 5%), Finland (1/20, 5%), and Norway (1/20, 5%). The sample size in each of the studies ranged from 25-571 for a total of number 3789 subjects in all. The median follow-up time was 4 months with a range of 1 week to 18 months. Thirteen (65%) of the 20 trials, were conducted among patients with breast cancer [12,16-18,21,22,25-31], followed by four trials in patients with any solid cancer [13-15,23], and one trial each with focus on cervical [24], colorectal [19], and colorectal/prostate cancers [20].

Many of the included studies (14/20, 70%) were telephone-based interventions, although two of them were used in conjunction Web-based systems. Most of these telephone-based interventions (12/14, 85.7%) involved a professional interventionist (nurses, psychologists, or counselors) trained to provide counseling, while the remaining two studies [18,25] were delivered by peer counselors who are cancer survivors. Additionally, only one of these telephone-based studies utilized automated voice response [23], which was actually used in conjunction with life-support personnel. Five of the studies [15,27,29-31] used Web-based delivery systems for their interventions, and one study [28] utilized store-and-forward video-recorded sessions to deliver their intervention. The duration and frequency of the interventions varied and so also the total intervention time with a median of 12 weeks and range of 1 week to 12 months. Table 2 summarizes the main results from each of the studies showing effects of the intervention on primary outcomes.

**Table 2:** Results showing effects of the intervention on primary outcomes^a^.

Author, year, country	Follow-up time	Outcome	Outcome measurement	Effect measure	Effect size	*P* value
Kim, [14] 2013, Korea	1 week	Pain	BPI	Mean pain score; proportion with pain score ≥4	-0.3; -16%	.24; .02
Rustoen, [13] 2013, Norway	6 weeks	Pain	Numerical rating scale	Mean change in pain intensity score	No effect	NS
Kroenke, [23] 2010, USA	12 months	Pain, depression	BPI, HSCL-20	Mean difference	-0.70; -0.26	<.001; <.001
Badger, [12] 2013, USA	16 weeks	Depression	CES-D	Mean difference	No effect	NS
Borosund, [27] 2014, Sweden	6 months	Depression	HADS	Mean difference compared with control	Webchoice: -0.79; IPPC: 0.69	.03; .03
Duffecy, [15] 2013, USA	8 weeks	Depression	HADS	Mean difference	0.26	--
Gotay, [25] 2007, USA	3 months	Depression	CES-D	Odds ratio of proportion with scores ≥16	1.38	.24
Lepore, [29] 2014, USA	1 month	Depression	HADS	Unstandardized regression coefficients (S-ISG=0, P-ISG=1)	1.11	.028
Livingston, [20] 2010, Australia	12 months	Depression	HADS	Mean difference	0.16; -0.19	.55; .57
Marcus, [21] 2010, USA	18 months	Depression	CES-D	Mean difference; Proportion with scores ≥16	No change in mean scores; 0.23	NS; .06
Stanton, [30] 2013, USA	6 months	Depression	CES-D	Adjusted group means	5.8	.009
Freeman, [28] 2014, USA	3 months	HR-QOL	SF-36; FACT-B	Adjusted group means	Comparing LD vs TD vs WL: SF-36 PCS: 48.32 vs 49.93 vs 46.81; SF-36 MCS: 48.77 vs 49.40 vs 44.30 FACT-B: 24.66 vs 26.03 vs 23.66	.15; .02; .08
Harrison, [19] 2011, Australia	6 months	HR-QOL	FACT-C	Mean difference	7.4	.19
Hawkins, [22] 2010, USA	6 weeks	HR-QOL	WHOQOL	Mean difference	0.26, 0.19, 0.24	All <.05
Loprinzi, [18] 2011, USA	12 weeks	HR-QOL	LASA QOL	Mean difference	2.3	–
Nelson, [24] 2008, USA	4 months	HR-QOL	FACT-Cx	Mean difference	11.57	.012
Park, [16] 2012, Korea	3 months	HR-QOL	FACT-B	Mean difference	-17.18	.002
Ryhanen, [31] 2013, Finland	Throughout treatment period, average 9 months	HR-QOL	Quality of life instrument - breast cancer patient version	Mean QOL scores (ANOVA)		.82
Sandgren, [26] 2007, USA	13 months	HR-QOL	FACT-G	Mean score	96.84 vs 95.50 vs 97.00	>.11
Sherman, [17] 2012, USA	Phase-specific: within 14 days of completion of adjuvant chemotherapy or 6 months post-surgery	Psychological well-being	PAL-C	Mean change	No effect	NS

^a^HR-QOL: Health-related Quality of Life; CES-D: Center for Epidemiological Studies-Depression Scale; BPI: Brief Pain Inventory; HADS: Hospital Anxiety and Depression Scale; FACT-B: Functional Assessment of Cancer Therapy-Breast; PAL-C: Profile of Adaptation to Life Clinical Scale; LASA QOL: Linear Analog Self-Assessment Quality of Life; FACT-C: Functional Assessment of Cancer Therapy-Colorectal; WHOQOL: World Health Organization Quality of Life; HSCL-20: 20-item Hopkins Symptom Checklist; FACT-Cx: Functional Assessment of Cancer Therapy-Cervical; FACT-G: Functional Assessment of Cancer Therapy Scale-General; NS: non-significant.

**Figure 2:**
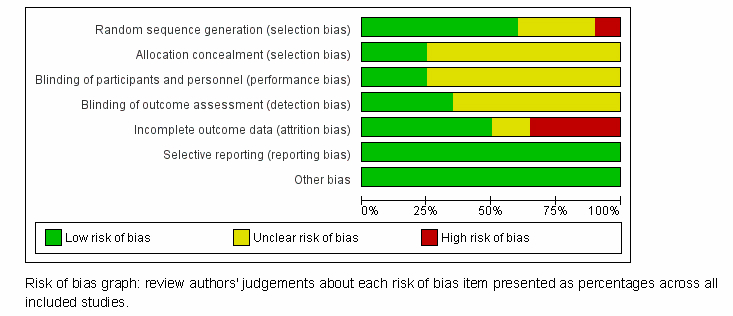
Risk of bias graph for included studies.

Figure 2 depicts the methodological quality of the studies included in this review. Most of the studies provided information about the method of generation of random sequence, while two studies [14,20] applied inappropriate methods of random sequence generation. Only a few studies (25%, 5/20) provided information about allocation concealment. Similarly, only a few studies (25%, 5/20) [15,17,23,29,31] had low risk of bias on the blinding of subjects and study personnel domain. Seven studies [13,15,18,21,22,24,27] were judged to have a risk of bias on the incomplete outcome reporting because of imbalance in dropout rates by group or insufficient accounting of all study participants.

Kim et al [14] compared the efficacy of pain education alone (control arm) and pain education plus telemonitoring (experimental arm) on pain and depression in a total of 108 patients with advanced solid tumors. In their trial, nursing specialists provided video-assisted educational material in both arms and daily telemonitoring for the first week in the experimental arm. There was significant improvement in pain and depression outcomes comparing baseline and final outcome in all study participants. They also reported significant reductions in number of intervention subjects with pain intensity scores ≥4 compared with control group (35%-19%, *P*=.02). Although average pain score over the past 24 hours (-1.2 vs -1.9) and worst pain scores (-0.7 vs -1.9) decreased compared to control group, these were not significant. Similarly, Harrison et al [19] evaluated the effectiveness of a nurse-delivered telephone supportive intervention (the “CONNECT” intervention) compared with usual care in 75 colorectal cancer patients. The CONNECT intervention consisted of five calls from a specialist nurse in the 6 months after initial discharge from the hospital [32]. They also found time-dependent improvement in HR-QOL within each arm but failed to reach a statistical significance comparing intervention and control groups.

Livingston et al [20] enrolled 571 newly diagnosed male CRC (n=182) and prostate (n=389) cancer patients and randomized them into three arms: two intervention arms and a passive referral arm. In the active referral arms, the specialist actively referred men to a Cancer Helpline. In Active Referral-4, patients received calls from the Helpline within 1 week of diagnosis, at 6 weeks, 3 months, and 6 months post diagnosis. In the Active Referral-1 arm, patients received only one call within 1 week of diagnosis. In the control arm, Passive Referral, patients were referred to contact the Helpline at their own initiative. The telephone helplines were developed by many cancer organizations in Australia to provide information tailored to the cancer patient’s needs, support, and referral to supportive service [33]. The study included only male patients based on prior work that suggested that men were less likely to utilize supportive services [33,34]. However, they found no psychological impact of the telephone-based intervention; mean changes over time in cancer-specific depression outcomes were similar between study arms.

In 2010, Kroenke et al reported the results of the Indiana Cancer Pain and Depression (INCPAD) trial [23]. In this trial, 202 patients were randomly assigned to receive the intervention and 203 to receive usual care. Patients in the intervention group received centralized telecare management by a nurse-physician specialist team coupled with automated home-based symptom monitoring by interactive voice recording or online. They reported that the intervention resulted in improved pain and depression outcomes in cancer patients assigned to receive the intervention. The standardized effect size for between group differences at 3 and 12 months was 0.67 (95% CI 0.33-1.02) and 0.39 (95% CI 0.01-0.77) for pain, and 0.42 (95% CI 0.16-0.69) and 0.41 (95% CI 0.08-0.72) for depression.

## Discussion

### Principal Findings

This systematic review of randomized controlled trials evaluating the effect of telehealth interventions on pain, depression, and HR-QOL outcomes in cancer care included 20 studies published over a 9-year period from 2006-2014. From this review, we make a number of observations. First, the application of telehealth in cancer care is in early stages, and all the studies were conducted in high-income countries. Second, the studies were largely heterogeneous in design and outcome assessments, making it difficult to pool effects in a meta-analysis. Third, the interventions are diverse in terms of type, content, intervention times, follow-up periods, and outcome measures.

Two of the three studies included in this review that focused on pain control reported a positive effect of telehealth on improving outcomes [14,23]. However, the study by Rustoen et al attributed inadequate dose of the psychoeducational intervention as one of the probable reasons for lack of efficacy. This is in contrast to the other two studies that relied heavily on collaborative care management between patients, their caregivers, and health care providers with extensive patient education. The INCPAD trial also included automated symptom monitoring as part of the intervention. Previous systematic reviews have identified patient education as a key component in improving cancer pain management. Lovell et al proposed four core principles that should guide the basis for patient education to successfully improve cancer pain management. These include the principles that education should be (1) patient-centered, (2) be an integral component of the patient-provider relationship, (3) aimed at patient empowerment for self-management, and (4) incorporated as part of ongoing care to counsel and support patients in the context of the severity of their pain, their needs and self-management plans. Therefore, incorporating collaborative patient-centered psychoeducational strategies in the design of technology-based interventions for pain management could improve efficacy of technology-based pain management interventions.

In contrast to the pain-focused studies, three of the eight studies evaluating the effect of telehealth on HR-QOL demonstrated improved outcomes. A recent review by Dickson et al evaluating the use of technology-based interventions for cancer follow-up surmised that the interventions did not decrease HR-QOL [35]. Similarly, four of the ten studies evaluating the effect of telehealth interventions on depression demonstrated significant improvement in depression outcomes. The SMART oncology trial, another collaborative care management approach that was delivered by a care manager under the supervision of a psychiatrist, demonstrated improvement in depression outcomes [36]. Also, a meta-analysis evaluating the effect of various interventions on depression in cancer patients showed that compared with controls, psychotherapy significantly improved outcomes and cognitive behavioral therapy was particularly associated with better outcomes [37].

It is noteworthy that the majority of interventions reported in this review were telephone-based. This is unsurprising because telephone systems are one of the oldest and most reliable information technologies available today, which makes them a very popular communication tool across different generations. They also enable personal live interactions between patients and providers, which can enhance the patient’s sense of being supported. While they are also very cheap, the cost of maintaining a professional to deliver care coupled with the fact that treatment effects could be provider-dependent may hinder scalability. While there are no doubts that the current dominance of telephone-based interventions will continue, current trends suggest that mobile phones will be the primary medium of delivery. This is evidenced by the near global ubiquity of cellular coverage and the increasing affordability, portability, and ease of use of smartphones [38]. Current estimates suggest that about 56% of US adults own a smartphone, and we envisage that this upward trend will continue [39]. While the telephone-based interventions in this review are largely voice communications, text messaging now appears to be a dominant function that is being used to engage patients across multiple disease groups.

Although not specific to connected health-related studies, previous studies have highlighted similar challenge [40]. Okuyama et al in their review of clinical trials evaluating psychosocial telephone interventions in patients with cancers and survivors also reported a similar finding that the majority of the studies reviewed lacked a standardization of outcome assessments and did not adhere adequately to reporting according to CONSORT guidelines. To standardize the reporting of technology-based interventions, the CONSORT-EHEALTH Group developed checklists to provide useful guidelines in reporting technology-based trials [41]. The time is ripe to capitalize on the current optimism of the potential of telehealth to transform care delivery. To realize this goal, we cannot overemphasize the need to design high-quality trials to comprehensively establish evidence of the effectiveness of telehealth-related studies.

### Limitations

This study is not without limitations. The fact that we included only studies reported in English could have led to the exclusion of relevant studies, but we do not believe this will significantly impact our findings. We limited our search to five databases and also did not search the gray literature to find relevant studies nor did we include non-randomized, retrospective studies. We believe that evidence from prospective randomized trials will be sufficient to demonstrate effectiveness. In addition, there was a heterogeneity of outcomes, outcomes assessment measures, and comparators, which makes it difficult to estimate overall effects.

### Conclusions

This is one of the first studies seeking to evaluate the effect of telehealth on pain, depression, and quality of life outcomes in patients at different stages of their cancer experience. While the studies evaluating cancer pain outcomes proved to be effective, the same could not be reported for those evaluating depression and quality of life outcomes. In total, our findings suggest that the application of telehealth in cancer care is still at a very early stage and is mostly utilized in developed nations. Evidence of its effectiveness demonstrates promise of improving pain, depression, and HR-QOL-related outcomes in cancer patients. There is a need to invest resources into developing rigorous larger-sized clinical trials, standardize outcome assessments, and improve reporting of clinical trials to demonstrate the effect of telehealth and realize the potential of transforming care delivery.

## Abbreviations

BPI: Brief Pain Inventory
CES-D: Center for Epidemiological Studies-Depression Scale
CHESS: Comprehensive Health Enhancement Support System
CRC: colorectal carcinoma
FACT-B: Functional Assessment of Cancer Therapy-Breast
FACT-C: Functional Assessment of Cancer Therapy-Colorectal
FACT-Cx: Functional Assessment of Cancer Therapy-Cervical
FACT-G: Functional Assessment of Cancer Therapy Scale-General
HADS: Hospital Anxiety and Depression Scale
HR-QOL: Health-Related Quality of Life
HSCL-20: 20-item Hopkins Symptom Checklist
INCPAD: Indiana Cancer Pain and Depression trial
LASA QOL: Linear Analog Self Assessment Quality of Life
NCCN: National Comprehensive Cancer Network
NCI: National Cancer Institute
NP: nurse practitioner
PAL-C: Profile of Adaptation to Life Clinical Scale
PHQ-9: Patient Health Questionnaire-9
RCT: randomized controlled trial
SMART: Stress Management and Resiliency Training
WHO: World Health Organization
WHOQOL: World Health Organization Quality of Life
